# Vincamine alleviates intrahepatic cholestasis in rats through modulation of NF-kB/PDGF/klf6/PPARγ and PI3K/Akt pathways

**DOI:** 10.1007/s00210-024-03119-2

**Published:** 2024-05-18

**Authors:** Rania Alaaeldin, Yusra A. Eisa, Mahmoud A. El-Rehany, Moustafa Fathy

**Affiliations:** 1 Department of Biochemistry, Faculty of Pharmacy, Deraya University, Minia, 61111 Egypt; 2 Department of Biochemistry, Faculty of Pharmacy, Minia University, Minia, 61519 Egypt

**Keywords:** Vincamine, Intrahepatic cholestasis, Oxidative stress, NF-kB, PPARγ, PI3K/Akt, Apoptosis

## Abstract

The defect in the hepatobiliary transport system results in an impairment of bile flow, leading to accumulation of toxic compounds with subsequent liver disorders. Vincamine, a plant indole alkaloid that is utilized as a dietary supplement, has been known for its promising pharmacological activities. For the first time, the present study was planned to estimate, at the molecular level, the potentiality of vincamine against alfa-naphthyl isothiocyanate (ANIT)-induced hepatic cholestasis. Liver function tests were analyzed. Hepatic activity of SOD and levels of GSH and MDA were assessed. Hepatic contents of bax, bcl2, NF-kB, PPARγ, catalase, heme-oxygenase-1, NTCP, and BSEP were evaluated using ELISA. mRNA levels of *NF-kB, IL-1β, IL-6, TNFα, PDGF, klf6, PPARγ*, and *P53* were examined using qRT-PCR. PI3K, Akt and cleaved caspase-3 proteins were assessed using western blotting. Histopathological analyses were performed using hematoxylin & eosin staining. ANIT-induced hepatic cholestasis elevated liver function tests, including AST, ALT, GGT, ALP, and total bilirubin. ANIT reduced the protein expression of NTCP and BSEP hepatic transporters. It induced the expression of the inflammatory genes, *TNFα, IL-6, IL-1β*, and *PDGF*, and the expression of NF-kB at the genetic and protein level and suppressed the anti-inflammatory genes, klf6 and *PPARγ*. Also, antioxidant markers were reduced during ANIT induction such as GSH, SOD, catalase, heme-oxygenase-1 and PI3K/Akt pathway, while MDA levels were elevated. Furthermore, the expression of *P53* gene, bax and cleaved caspase 3 proteins were activated, while bcl2 was inhibited. Also, the histopathological analysis showed degeneration of hepatocytes and inflammatory cellular infiltrates. However, vincamine treatment modulated all these markers. It improved liver function tests. It inhibited the expression of *NF-kB, TNFα, IL-6, IL-1β* and *PDGF* and activated the expression of *klf6* and *PPARγ*. Furthermore, vincamine reduced MDA levels and induced GSH, SOD, catalase, heme-oxygenase-1 and PI3K/Akt pathway. Additionally, it inhibited expression of *P53* gene, bax and cleaved caspase 3 proteins. More interestingly, vincamine showed better outcomes on the hepatic histopathological analysis and improved the alterations induced by ANIT. Vincamine alleviated hepatic dysfunction during ANIT-induced intrahepatic cholestasis through its anti-inflammatory and antioxidant efficacies by the modulation of NF-kB/PDGF/klf6/PPARγ and PI3K/Akt pathways.

## Introduction

Liver is the largest solid organ in the body; it maintains normal blood glucose levels, removes toxins from the blood supply, and regulates blood clotting. One of the liver's primary functions is the formation of bile, a mixture of organic and inorganic compounds produced by cholangiocytes and hepatocytes to help digestion by exhibiting detergent properties that aid in lipid breakage into small particles (Boyer and Soroka 2021).

Hepatic cholestasis is a clinical condition that results from a reduction of bile flow and destruction of the normal bile excretory system (Ding et al. 2008). Several pathways and gene mutations contribute to hepatic cholestasis, including mutation in the bile acid transporter genes or the transcription factors required for bile formation (Alvarez et al. 2004). Additionally, viral hepatitis, cholestasis of pregnancy, auto-immune hepatitis, non-alcoholic liver disease, primary biliary cirrhosis, and drug-induced hepatic injury were reported to cause intra-hepatic cholestasis (Chen et al. 2016). If left untreated, cholestasis will develop jaundice and hypercholesterolemia, which may eventually cause hepatic fibrosis, cirrhosis, and hepatic failure (Boyer 2007).

Moreover, numerous studies reported the activation of the inflammatory response system and oxidative stress in hepatic cholestasis (Jaeschke 2011). The activation of inflammatory mediators results in modulation of the expression of different number of transcription regulators which further cause profound and rapid bile flow reduction (Kosters and Karpen 2010). Additionally, during hepatic cholestasis, neutrophils accumulate in the liver and subsequently evoke reactive oxygen species (ROS) to develop liver injury and oxidative stress (Copple et al. 2010).

Alfa-naphthyl isothiocyanate (ANIT) is a known compound that induces a dose-dependent acute intrahepatic cholestasis in the research field (Cui et al. 2009). ANIT administration has been one of the most extensive methods used to induce intrahepatic cholestasis (Rodríguez-Garay 2003). ANIT was reported to cause pathological and biochemical changes closely similar to hepatic cholestasis, including bile duct obstruction via cholangiolitic hepatitis, epithelial apoptosis or necrosis within the interlobular duct, neutrophil infiltration around bile ducts, damage of the epithelial cells and decelerating bile flow (Amin et al. 2006).

The goal is to anticipate the disease progression, reverse the bile duct inflammation, and stop the development of chronic cholestasis. All present therapy to treat hepatic cholestasis are only Budesonide, Fibrates, and Corticosteroids; however, they have not shown a complete satiated efficacy. Hence, screening for new therapeutic agents for hepatic cholestasis treatment is well deserved.

Vincamine is a well-known naturally occurring indole alkaloid compound extracted from the leaves of vinca minor and has been widely studied since the 1950s (Patangrao Renushe et al. 2022). It has several pharmacological actions, including antioxidant, anti-inflammatory, anti-apoptotic, lung protective, hepatoprotective, and nephroprotective activities (Wu et al. 2018; Fawzy et al. 2022; Renushe et al. 2022; Alaaeldin et al. 2023). Therefore, for the first time, the present study is prompted to investigate, at the molecular level, the effect of vincamine against ANIT-induced intrahepatic cholestasis.

## Materials and methods

### Drugs and chemicals

Vincamine was obtained from Sigma Aldrich (#1617–90-9, Sigma-Aldrich, Inc, St Louis, MO, USA). Carboxymethyl cellulose (CMC, 0.5%) was used for the administration of vincamine.

### Experimental animals

Animal care and study protocols were followed according to the guidelines approved by The Research Ethics Committee and Experimental Animal Center, Minia University, Minia, Egypt (Approval No: ES09/2021). Adult male Wistar rats (180–200 g and 7–8 weeks' old) were purchased from Deraya University's Animal Research Center. For animal housing, separate cages were utilized supplied with commercial pellets for feeding and fresh drinking water and conserved in a natural condition (12 h of light/dark cycles) during the experiment.

### Induction of intrahepatic cholestasis and experimental design

Intrahepatic cholestasis was induced in animals using a single dose of ANIT (60 mg/kg; P·O.)(Fawzy et al. 2023). 0.5% CMC or vincamine treatment was administrated to different groups from day 1 in a similar manner. The experiment lasted for 10 days, whereas ANIT was administrated at the 8th day and the animals were sacrificed 48 h later (Fawzy et al. 2023). Blood samples and liver tissues were obtained. Liver tissue was split into three parts: the first part, for the histological investigations, was conserved in 10% neutral buffered formaldehyde, the other two parts were directly stored at – 80° C for further RNA and protein assessment.

### Experimental design

A total of 30 rats were assigned at random into five groups as follows:

- Group I (control group, *n* = 6): 0.5% CMC was administrated orally from day 1 to day 10.
- Group II (Vincamine group, *n* = 6): Animals received oral administration (40 mg/kg) of vincamine dissolved in 0.5% CMC daily from day 1 till the end of the experiment (El-Sayed et al. 2021).
- Group III (ANIT group, *n* = 6): Animals received a single dose of ANIT (60 mg/kg; P·O.)(Fawzy et al. 2023) at the 8th day and orally administrated 0.5% CMC from day 1 to day 10.
- Group IV (ANIT/Vincamine, *n* = 6): Animals received a single dose of ANIT (60 mg/kg; P·O.) at the 8th day and orally administrated vincamine (40 mg/kg) daily from day 1 to day 10.
- Group V (ANIT/Quercetin group, *n* = 6): This group was used as a positive control group in which animals received a single dose of ANIT (60 mg/kg; P·O.) at the 8th day and orally administrated quercetin (50 mg/kg) daily from day 1 to day 10 (Li et al. 2016).

### Evaluation of liver function

Serum levels of alanine transaminase (ALT) (#12,212, Human, Wiesbaden, Germany), (aspartate transaminase (AST) (#12,211, Human, Wiesbaden, Germany), alkaline phosphatase (ALP) (#12,117, Human, Wiesbaden, Germany), gamma-glutamyl transferase (GGT) (#12,213, Human, Wiesbaden, Germany), and total bilirubin (#10,740, Wiesbaden, Germany) were examined utilizing their corresponding kits according to manufacturer’s instructions.

### Assessment of hepatic antioxidant and oxidative stress status

To evaluate the oxidative stress markers in cholestatic groups with or without vincamine treatment, hepatic tissue activity of superoxide dismutase (SOD) and levels of reduced glutathione (GSH) and malondialdehyde (MDA) were evaluated utilizing (#SD2521, Biodiagnostic, Gizza Egypt), (#GR2511, Biodiagnostic, Gizza Egypt), (#MD2529, Biodiagnostic, Gizza Egypt), respectively.

## ELISA technique

Hepatic content of NF-kB protein was assessed using Rat NFKB-p65 (Nuclear Factor Kappa B p65) ELISA Kit (#E-EL-R0674, Elabscience, USA). Hepatic PPARγ content was measured using Rat PPAR-γ (Peroxisome Proliferator Activated Receptor Gamma) ELISA Kit (#MBS2508012, MyBioSource, CA, USA). Hepatic content of catalase was assessed using Rat Catalase ELISA Kit (#KT-9509, Kamiya Biomedical Company, WA, USA). Hepatic content of heme-oxygenase-1 (HO-1) was evaluated using Rat Heme Oxygenase 1 SimpleStep ELISA® Kit (#ab279414, Abcam, Cambridge, UK). The hepatic transporter sodium taurocholate co-transporting polypeptide (NTCP) protein was measured utilizing Rat Na + Taurocholate Cotransporting Polypeptide ELISA Kit (#MBS3809890, MyBioSource, CA, USA), while the hepatic transporter bile salt export pump (BSEP) protein was assessed using Rat Bile salt export pump (ABCB11) Elisa kit (Competitive ELISA) (#MBS7215031, MyBioSource, CA, USA). Hepatic levels of bax and bcl2 proteins were evaluated utilizing Rat bax ELISA kit (#LS-F21494, LifeSpan BioSciences, MA, USA) and rat bcl2 ELISA kit (#MBS704330, MyBioSource, CA, USA), respectively, according to manufacturer’s instructions.

### Quantitative real-time PCR

Total RNA was extracted from liver tissue samples according to the Qiagen RNA extraction kit (Hilden, Germany) instructions. The expression of *nuclear factor kappa B (NF-κB), tumour necrosis factor α (TNF-α), IL-6, IL-1β, p53, platelet derived growth factor (PDGF), Peroxisome proliferators–activated receptor γ (PPAR γ),* and *Krüppel-like factor 6 (klf6)* genes was assessed by real-time qPCR. Using the Rotor-Gene 6000 Series Software 1.7, mRNA quantification was got. As an internal control, *Glyceraldehyde 3-phosphate dehydrogenase (GAPDH)* was utilized (Barber et al. 2005). The sequences of the used primers are mentioned in Table 1, primer sequences were obtained from National Centre for Biotechnology Information (NCBI). Using the Qiagen one step RT-PCR (Qiagen), RT-PCR reactions were implemented, containing 1 × buffer, forward and reverse primers (0.6 μM), 100 ng of total RNA, enzyme mix (2 μl), and 400 μM each of dNTP. The conditions were as follows: 35 cycles of denaturation step at 95ºC (25 Sec), primers annealing at 58 ºC (30 Sec), and polymerization step at 72 ºC (20 Sec).

**Table 1:** Sequences of the used primers

Primer	Sequence
*NF-κB*	F: 5´- TCAACATGGCAGACGACGATCC -3´ R: 5´- GAAGGTATGGGCCATCTGTTGAC -3´
*IL6*	F: 5´-CCTACCCCAACTTCCAATGCT -3´ R: 5´-GGTCTTGGTCCTTAGCCACT -3´
*TNFα*	F: 5´- GGAGGGAGAACAGCAACTCC-3´ R: 5´- GCCAGTGTATGAGAGGGACG -3´
*IL-1β*	F: 5´- CCTATGTCTTGCCCGTGGAG -3´ R: 5´- CACACACTAGCAGGTCGTCA -3´
*P53*	F: 5´- AGCGACTACAGTTAGGGGGT -3´ R: 5´- ACAGTTATCCAGTCTTCAGGGG -3´
*Klf6*	F: 5´- CCCTGCTGTGTGCTTATCCA -3´ R: 5´- AGGCATTGACACATACGGCA -3´
*PDGF*	F: 5´- CAGTCCCGGCTACCCTATCT -3´ R: 5´- GCCCCTCCTCACTCCAAAAG -3´
*PPAR γ*	F: 5´- GCTGTTATGGGTGAAACTCTGG -3´ R: 5´- ATAGGCAGTGCATCAGCGAA -3´
*GAPDH*	F: 5´- CTCTCTGCTCCTCCCTGTTC -3´ R: 5´- CGACATACTCAGCACCAGCA -3´

RT-PCR reactions were implemented in triplicate for each sample. For each sample, the average cycle threshold (Ct) was estimated. Using the SYBR Green fluorescent dye, to depict the acquired amplified mixture with the revocation of contamination and to eliminate the generation of non-specific compounds, a melting curve analysis was obtained at 1˚C intervals between 60– 95˚C using the Rotor-Gene 6000 Series Software 1.7. Relative to the untreated sham group, the different genes expression in the treated groups, after *GAPDH* expression normalization, was obtained.

### Western blotting analysis

Sodium dodecyl sulphate–polyacrylamide gel electrophoresis (SDS-PAGE) analysis was performed to detect the expression of phosphorylated and total phosphoinositide-3-kinase (PI3K), protein kinase B (Akt), and cleaved caspase-3 proteins.

Protein was extracted from liver tissue samples using RIPA lysis buffer, containing NaCl (150 mM), SDS (0.1%), Tris–Cl (50 mM), pH 7.5; sodium deoxycholate (0.5%), Nonidet P-40 (1%), and PMSF (1 mM), boosted with the complete protease inhibitor cocktail (Roche, Mannheim, Germany). For estimation of the protein concentration, Bradford method was used (Bradford 1976). By SDS-PAGE (15%), cell lysates (30 μg protein) were separated and incubated in Blocking Solution at room temperature for 1 h after the transfer to Hybond™ nylon membrane (GE Healthcare). Then, at 4˚C, they were incubated overnight with antibodies of pPI3K (#phospho Y607, ab182651, abcam, Cambridge, UK), PI3K (#ab154598, abcam, Cambridge, UK), pAkt (#phospho T308 ab38449, abcam, Cambridge, UK), Akt (#ab8805, abcam, Cambridge, UK), and cleaved caspase-3 (#ab214430, abcam, Cambridge, UK) diluted (1:1000) with PBS. Then, membranes, for 15 min, were washed and incubated with the HRP-conjugated secondary antibody (abcam, Cambridge, UK) for 1 h at room temperature, diluted (1:1000) in PBS (Greenfield 2013). By a luminescent image analyzer (LAS-4000, Fujifilm Co., Tokyo, Japan), immunoreactive proteins were estimated, according to the manufacturer’s instructions, using an enhanced chemiluminescence kit (GE Healthcare, Little Chalfont, UK). Antibody against β-actin (#sc47778, Santa Cruz Biotechnology, Santa Cruz, CA) (1:1000) was used to detect β-actin. Using Bio-Rad Trans-Blot SD Cell apparatus (Bio-Rad, Hercules, CA, USA), electroblotting and electrophoresis, with a discontinuous buffer system, were performed. By The Image Processing and Analysis Java (ImageJ, 1.8.0_172) program, densitometric analysis was then carried out after normalization to the corresponding β-actin levels and data were expressed relative to the untreated sham group.

### Histological analysis

Liver tissue sections were fixed with 10% formaldehyde, then, dehydrated in ascending grades of ethanol and embedded in paraffin (4 µm for thickness). Then, the sections were stained with hematoxylin–eosin (H&E) and observed for histopathological changes using the light microscope (Olympus, Tokyo, Japan) and using a high-quality digital camera mounted on the microscope, photographs were taken digitally. The assessment was carried out in a blinded manner. Histopathological changes in each liver were quantified for the assessment of hepatic damage, in consideration of the area of necrosis, degeneration (ballooning), and foci of inflammation. Scoring of tissue injury was expressed as follows: 0; normal tissue, 1; mild tissue damage (< 10% of hepatocytes), 2; moderate tissue damage (10–50% of hepatocytes), 3; marked tissue damage (> 50% of hepatocytes) (Li et al. 2016).

### Statistical analysis

Data were expressed as mean ± standard deviation (SD). To analyze the differences of multiple comparison, one or two-way analysis of variance (ANOVA) followed by post hoc Tukey–Kramer test were performed using Excel software (Microsoft, Redwood, WA, USA) and GraphPad Prism 9 statistical software (GraphPad, La Jolla, CA, USA). When probability values (P) < 0.05, differences were deemed significant.

## Results

### Liver function tests

**Fig. 1:**
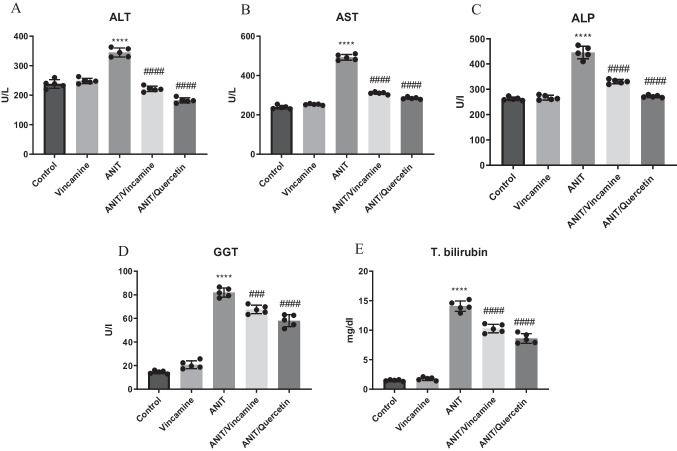
Serum levels of ALT (**A**), AST (**B**), ALP (**C**), GGT (**D**), and T.bilirubin (**E**) in different groups. Bars represent mean ± SD. Significant difference was analyzed by one-way ANOVA test followed by Tukey–Kramer multiple comparisons post hoc test, where ****; *P* < 0.0001, compared to sham group, and ###; *P* < 0.001, ####; *P* < 0.0001, compared to ANIT group

ANIT induction significantly (*P* < 0.0001) elevated serum levels of ALT, AST, ALP, GGT and total bilirubin to 345.0 ± 15.27 U/L, 492.6 ± 14.46 U/L, 445.5 ± 24.73 U/l, 81.94 ± 3.87 U/l, and 14.08 ± 0.87 mg/dl, respectively, compared to sham group. After vincamine treatment, serum levels of ALT, AST, ALP, and total bilirubin significantly (*P* < 0.0001) decreased to 221.5 ± 8.98 U/L, 310.5 ± 5.94 U/L, 330.6 ± 8.23 U/l, and 10.28 ± 0.719 mg/dl, respectively, compared to ANIT group. whereas GGT levels showed notable decrease (*P* < 0.001) to 67.64 ± 3.56 U/l, compared to ANIT group, as shown in Fig. 1. Quercetin was used as a positive control.

### Effect of vincamine on hepatic SOD activity and levels of MDA and GSH

**Fig. 2:**
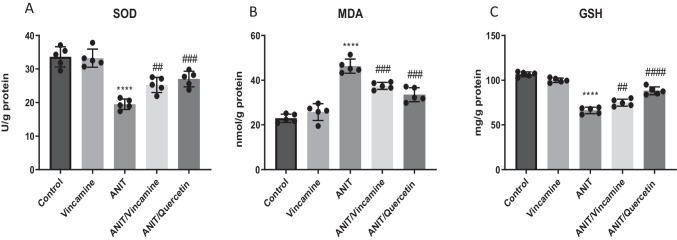
Hepatic tissue levels of SOD (**A**), MDA (**B**), and GSH (**C**) in different groups. Bars represent mean ± SD. Significant difference was analyzed by one-way ANOVA test followed by Tukey–Kramer multiple comparisons post hoc test, where ***; *P* < 0.001, ****; *P* < 0.0001, compared to sham group, and ##; *P* < 0.01, ###; *P* < 0.001, ####; *P* < 0.0001, compared to ANIT group

To evaluate the oxidative stress and oxidant conditions during ANIT induction with or without vincamine treatment, hepatic tissue SOD activity and levels of GSH and MDA were examined. As shown in Fig. 2A-C, ANIT-induced cholestasis significantly decreased (*P* < 0.0001) tissue levels of SOD and GSH to 19.5 ± 1.55 U/g and 66.18 ± 3.59 mg/g, respectively, compared to sham group. After vincamine treatment, SOD and GSH tissue levels were significantly elevated (*P* < 0.01) to 25.26 ± 2.27 U/g and 74.92 ± 3.85 mg/g, respectively, compared to ANIT group. Regarding MDA tissue levels, ANIT induction significantly increased (*P* < 0.0001) MDA levels to 46.26 ± 3.15 nmol/g, compared to sham group, while after vincamine treatment, MDA levels were reduced (*P* < 0.001) to 37.46 ± 1.54 nmol/g, compared to ANIT group. Qurcetin was used as a positive control.

### Effect of vincamine on the hepatic transporters, NTCP and BSEP

**Fig. 3:**
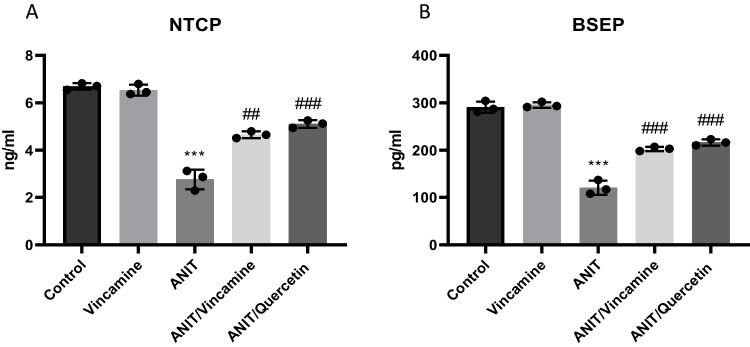
Protein levels of the hepatic transporters NTCP (**A**) and BSEP (**B**) in different groups. Bars represent mean ± SD. Significant difference was analyzed by one-way ANOVA test followed by Tukey–Kramer multiple comparisons post hoc test, where ***; *P* < 0.001, compared to sham group, and ##; *P* < 0.01, ###; *P* < 0.001, compared to ANIT group

To assess the protein expression levels of the hepatic transporters during ANIT induction with or without vincamine treatment, protein levels of the hepatic transporters NTCP and BSEP were measured. As shown in Fig. 3A, B, ANIT induction significantly reduced (*P* < 0.001) NTCP and BSEP protein levels to 2.76 ± 0.42 and 120.7 ± 14.84, respectively, compared to sham group. After vincamine treatment, NTCP and BSEP levels were significantly (*P* < 0.01 and *P* < 0.001) elevated to 4.653 ± 0.14 and 202.7 ± 4.50, respectively, compared to ANIT group.

### Effect of vincamine on hepatic bax and bcl2 proteins

**Fig. 4:**
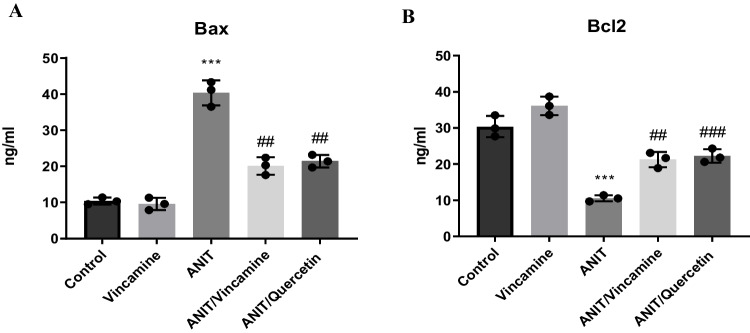
Hepatic tissue levels of Bax (**A**) and Bcl2 (**B**) in different groups. Bars represent mean ± SD. Significant difference was analyzed by one-way ANOVA test followed by Tukey–Kramer multiple comparisons post hoc test, where ***; *P* < 0.001, compared to sham group, and ##; *P* < 0.01, ###; *P* < 0.001, compared to ANIT group

To examine the apoptotic status of liver tissue during ANIT-induced cholestasis with or without vincamine treatment, hepatic tissue levels bax and bcl2 proteins were measured. As shown in Fig. 4**,** bax tissue levels were significantly (*P* < 0.001) increased to 40.33 ± 3.48 ng/ml during ANIT induction, compared to sham group, while following vincamine treatment, bax levels significantly decreased (*P* < 0.01) to 20.1 ± 2.40 ng/ml, compared to ANIT group. Whearas bcl2 tissue levels were significantly decreased (*P* < 0.001) to 10.53 ± 0.85 ng/ml, compared to sham group, but after vincamine treatment, bcl2 levels significantly increased (*P* < 0.01) to 21.23 ± 2.13 ng/ml, compared to ANIT group. While qurcetin was used as a positive control.

### Effect of vincamine on the expression of TNFα, IL-6, IL-1β, NF-kB, Klf6, PDGF, PPARγ, p53, catalase, and HO-1

To further evaluate the inflammatory state of liver tissue during ANIT-induced cholestasis, before and after vincamine treatment, mRNA levels of *TNFα*, *IL-6*, *IL-1β*, *NF-kB*, *Klf6*, *PDGF*, *PPARγ*, and *p53* were examined as well as the protein levels of the PPARγ target candidates, catalase and HO-1.

**Fig. 5:**
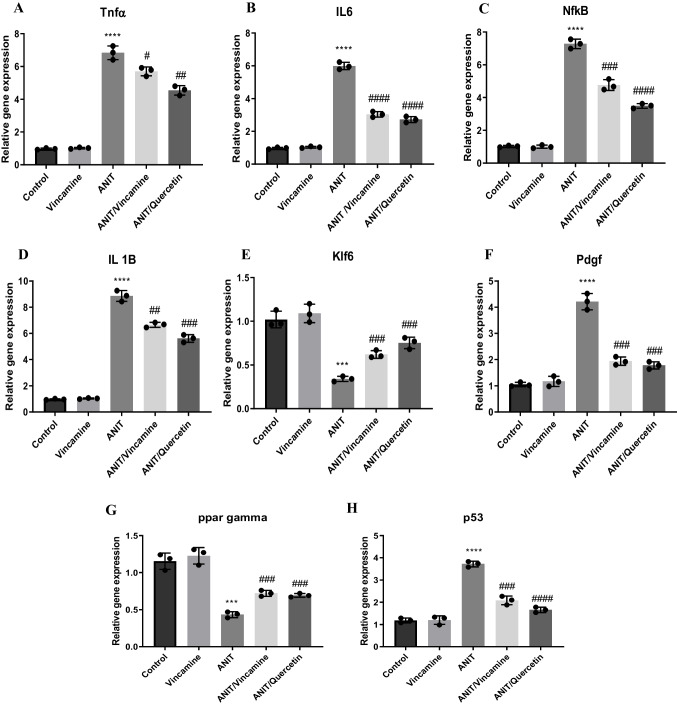

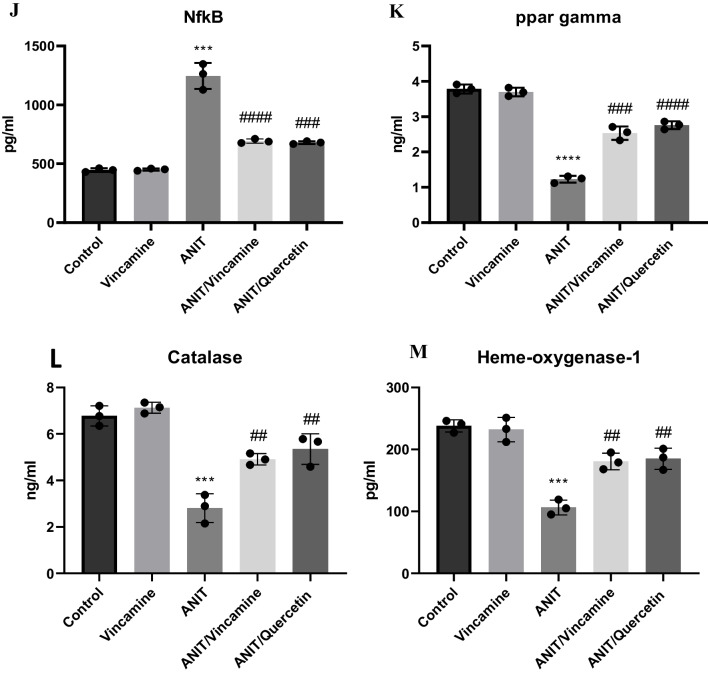
Expression of *TNFα* (**A**), *IL-6* (**B**), *NF-Kb* (**C**)*, IL-1β* (**D**), *Klf6* (**E**), *PDGF* (**F**), *PPARγ* (**G**) and *p53* (**H**) genes and protein levels of NF-kB (**J**), PPARγ (K), catalase (**L**), and HO-1 (**M**). qRT-PCR was measured within different groups. Expression was normalized to the corresponding *GAPDH* gene expression and presented relative to the untreated sham group. Bars represent mean ± SD. Significant difference was analyzed by one-way ANOVA followed by followed by Tukey–Kramer multiple comparisons post hoc test, where ***; *p* < 0.001, ****; *p* < 0.0001, compared to sham group, and #; *p* < 0.05, ##;*p* < 0.01, ###; *p* < 0.001, and ####; *p* < 0.0001, compared to ANIT group

As shown in Fig. 5, the mRNA levels of *TNFα*, *IL-6*, *IL-1β*, *NF-kB*, *PDGF*, and *p53* were significantly (*P* < 0.0001) upregulated in ANIT-induced group as well as *NF-kB* protein level, compared to sham group, while following vincamine treatment, their gene expression was notably decreased, compared to ANIT group. Whereas mRNA levels of klf6 and *PPARγ* were significantly suppressed (*P* < 0.001) as well as PPARγ protein level during ANIT induction, compared to sham group. However, after vincamine administration, their gene expression was notably (*P* < 0.001) elevated, so was *PPARγ* protein level, compared to ANIT group. Likewise, the protein levels of catalase and HO-1were significantly reduced (*p* < 0.001) in ANIT-induced group compared to sham group, while after vincamine treatment their protein levels significantly increased (*p* < 0.01) compared to ANIT group.

### Effect of vincamine on the expression of PI3K, AKT and c-caspase 3 proteins

**Fig. 6:**
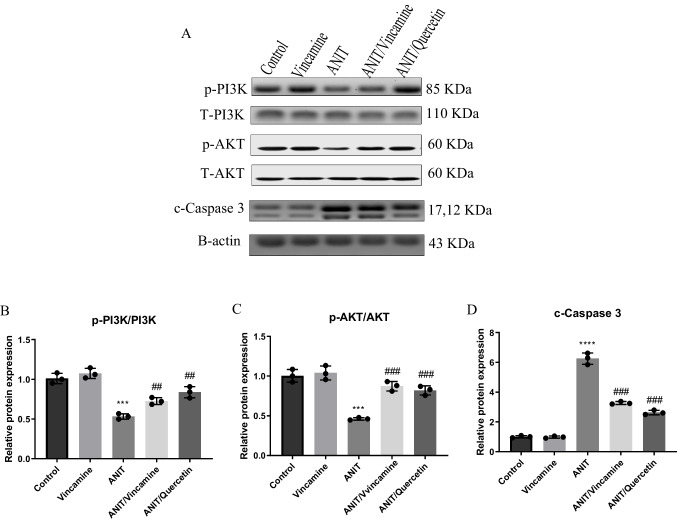
Effect of vincamine on the expression of PI3K, AKT, and c-caspase 3 proteins. (**A**) Representative western blots of PI3K, AKT, cleaved caspase 3 in different groups. Expression of phosphorylated/total PI3K (**B**), phosphorylated/total AKT (**C**), and c-caspase 3 (**D**) were expressed densitometrically, using bands in (A) after normalization to the corresponding internal control β-actin as fold change relative to that of sham control rats. Bars represent mean ± SD. Significant difference was analyzed by one-way ANOVA followed by followed by Tukey–Kramer multiple comparisons post hoc test, where ***; *p* < 0.001, ****;*p* < 0.0001, compared to sham group, and ##;*p* < 0.01, ###; *p* < 0.001, compared to ANIT group

The expression of phosphorylated and total PI3K and AKT in addition to the c-caspase 3 proteins was evaluated in the present study during ANIT induction with and without vincamine treatment. As shown in Fig. 6, the phosphorylated/total ratio of PI3K and AKT was considerably (*P* < 0.001) decreased during ANIT induction. However, vincamine treatment showed notable (*P* < 0.01 and *P* < 0.001) elevation for the phosphorylated/total ratio of PI3K and AKT, respectively, compared to ANIT group. On the other hand, c-caspase 3 protein expression was remarkably (*P* < 0.0001) elevated during ANIT induction, while vincamine administration exhibited significant decrease (*P* < 0.001) in c-caspase 3 protein expression, compared to ANIT group. While quercetin was used as a positive control.

### The histopathological investigation

**Fig. 7:**
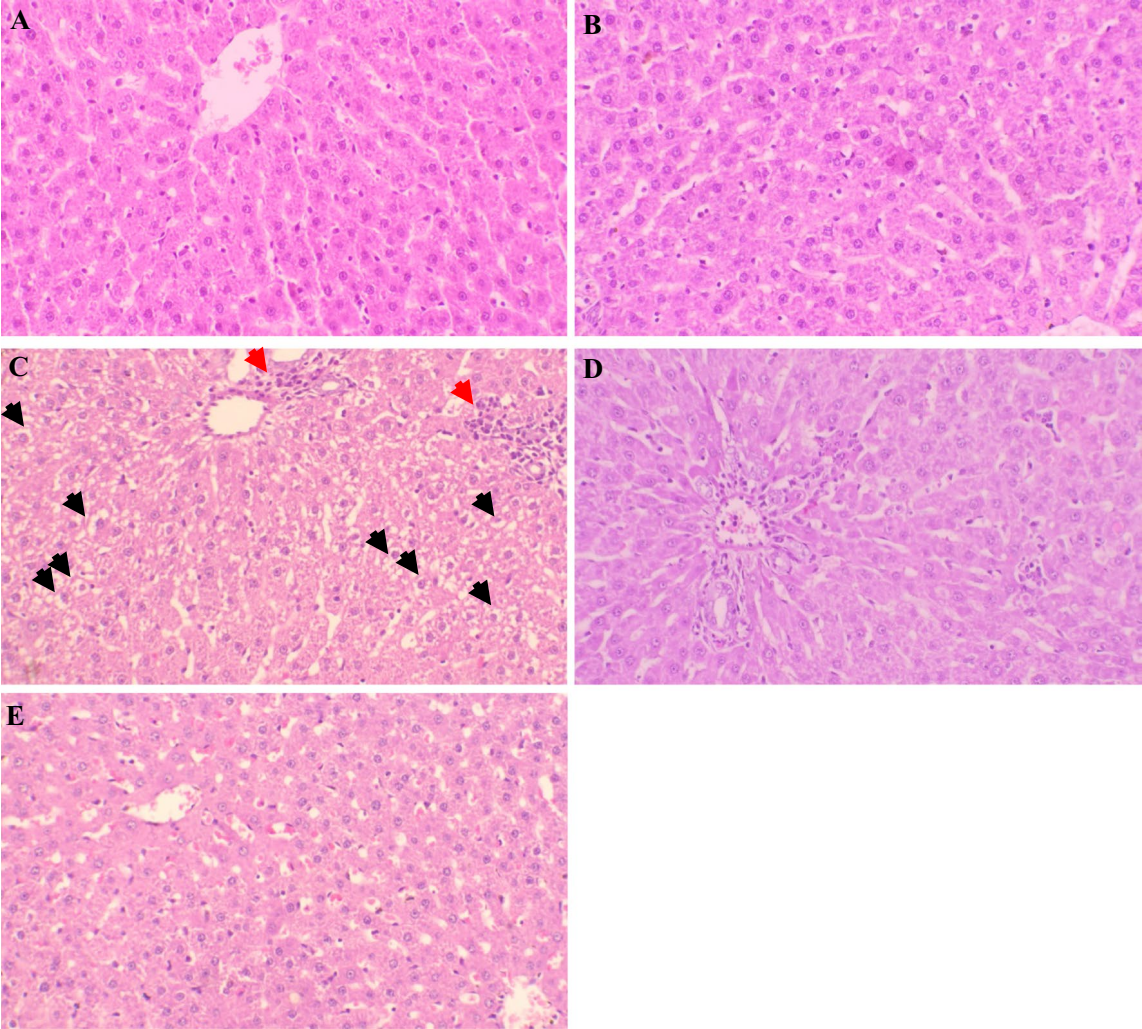
Representative photomicrographs of rat liver tissues of different groups. Liver tissues were stained with hematoxylin–eosin staining. (**A**) Sham group, (**B**) vincamine group, (**C**) ANIT group, (**D**) ANIT/vincamine group, and (**E**) ANIT/Quercetin group (magnification; × 200). (Black arrows); vacuolar degeneration of hepatocytes and (red arrows); inflammatory cellular infiltrates

Figure 5 and Table 2 demonstrate the histopathological alterations in the tissues and show the scoring of these histopathological alterations. As shown in Fig. 7A, hepatocytes showed normal enclosing blood sinusoids and normal portal tract. The vincamine control group showed normal appearing central vein and hepatocytes enclosing blood sinusoids (Fig. 7B). While ANIT-induced cholestasis group showed vacuolar degeneration of hepatocytes (black arrows) and inflammatory cellular infiltrates (red arrows) (Fig. 7C). However, following vincamine treatment, almost normal hepatocytes and central vein with scanty inflammatory cells were shown in the microphotographs (Fig. 7D). Additionally, Quercetin treatment demonstrated normal central vein, hepatocytes, and portal tract (Fig. 7E).

**Table 2:** Scoring of the histopathological tissues

Groups	Control	Vincamine	ANIT	ANIT/Vincamine	ANIT/Quercitin
Necrosis	0	0	0	0	0
Degeneration	0	0	2	0	0
Inflammation	0	0	3	1	0
Total scoring	0	0	5^**^	1^##^	0^##^

Significant difference was analyzed by one-way ANOVA followed by Tukey–Kramer multiple comparisons post hoc test, where **; *p* < 0.01, compared to sham group, and ##; *p* < 0.01, compared to ANIT group

## Discussion

Hepatic biliary uptake and excretion is facilitated by hepatobiliary transport system. A defect in the transport system would result in an impairment of bile flow, leading to accumulation of bile acids and toxic compounds with subsequent liver diseases, including hepatocellular carcinoma and fibrosis (Crocenzi et al. 2012; Fathy and Nikaido 2013; Abdelnaser et al. 2023). Physiological recruitment of alternative transporters to counteract the accumulation of toxic compounds is an adaptive solution for detoxifying pathways in the liver. Pro-inflammatory cytokines and bile acids mediate the adaptive transporter change through interactions with nuclear factors and receptors at the transcriptional level (Geier et al. 2007).

Due to the current unsatisfactory therapeutic agents, drug repurposing and screening agents for new pharmacological actions became essential (Alaaeldin et al. 2021a, 2021b, 2022; Sabra et al. 2022; Shytaj et al. 2022; Bekhit et al. 2023) whilst investigating the inflammatory and oxidative stress mechanisms underlying hepatic cholestasis could be a promising approach to better identify potential pharmacological strategies for management of hepatic cholestasis.

In the present study, against ANIT-induced intrahepatic hepatic cholestasis in rats, we examined, at the molecular level, the possible hepatoprotective activity of vincamine; a plant alkaloid that is utilized as a dietary supplement in the market and has been known for its promising antioxidant and anti-inflammatory activities (Al-Rashed et al. 2021; Patangrao Renushe et al. 2022).

Our initial assessment of liver functions was performed during ANIT induction with and without vincamine treatment through measuring the serum activities of liver associated enzymes, ALT, AST, ALP, and GGT, in addition to the levels of total bilirubin. Our findings imply that the serum activities of ALT, AST, ALP, GGT, and total bilirubin level were notably elevated during ANIT induction which indicate liver toxicity; however, administration of vincamine improved serum activities of ALT, AST, ALP, GGT, and total bilirubin level indicating the reduction of liver injury. This prompted further evaluation of vincamine activity against ANIT-induced hepatic cholestasis at the molecular level.

Bile acid transport is controlled by the hepatic transporters that regulate bile acid absorption and excretion homeostasis. However, cholestasis results in the dysregulation of bile acid transporters (Lam et al. 2010), therefore we assessed the effect of ANIT on the hepatic transporters and further studied the impact of vincamine treatment on ANIT-induced cholestasis. ANIT resulted in a notable decrease in the protein levels of the hepatic transporters NTCP and BSEP; however, administration of vincamine resulted in a significant increase in the protein levels of both NTCP and BSEP, indicating that vincamine restored the dysregulation of hepatic transporters caused by ANIT administration during cholestasis.

ANIT-induced hepatotoxicity was reported to cause neutrophil inflammation around bile duct and portal tract, and further evoke considerable hepatic inflammatory reaction, indicating that inflammation plays a critical role during hepatic cholestasis (Zhao et al. 2008). NF-kB is a primary mediator that transfers the inflammatory signals from the cytoplasm to the nucleus and further initiates the inflammatory signaling cascade in the cell such as TNFα, IL-6, and IL-1β (Akhtar et al. 2020; Fawzy et al. 2021; Zaki et al. 2022). Also, PDGF was reported to further activate NF-kB inflammatory signaling pathway (Nakamura et al. 2012). On the other hand, PPARγ and klf6 have different but related functions. Klf6, a transcription factor, controls the expression of genes linked to immune responses, modulating the inflammatory responses, including NF-kB-mediated inflammatory signaling cascade. In addition, its dysregulation may contribute to the amplification of the pro-inflammatory signals. Furthermore, the expression of the pro-inflammatory gene NF-kB is also suppressed by PPARγ, which is a nuclear receptor with anti-inflammatory capabilities. It has been observed that PPARγ activation is involved in the regulation of cell cycle, proliferation, apoptosis, and inflammatory pathways (Kim et al. 2019). Numerous studies reported that the activation of PPARγ attenuates inflammation through the induction of heme oxygenase-1 (HO-1) (Xu et al. 2015; Zhao et al. 2018). In addition, the elevated levels of HO-1 protect against excessive cell proliferation, fibrosis, and inflammation (Li et al. 2010). Additionally, one of the PPARγ target candidates to modulate oxidative stress and inflammation is the peroxisome-enriched antioxidant enzyme, catalase enzyme (Zhao et al. 2006). Geoffrey et al. indicated that administration of PPARγ ligands led to increase in catalase mRNA level and enzyme activity which eventually would lead to the activation to the antioxidant and anti-inflammatory defense mechanisms (Girnun et al. 2002).

In the present study, mRNA and protein levels of *NF-kB* and *PPARγ*, as well as mRNA levels of *IL-1β*, *IL-6*, *TNFα*, *klf6* and *PDGF* were evaluated, The expression *NF-kB*, *IL-1β*, *IL-6*, *TNFα* and *PDGF* genes were up-regulated by ANIT induction while the gene expression and protein level of *PPARγ* and gene expression of *klf6* gene were suppressed indicating the activation of the NF-kB-mediated inflammatory signaling pathway. However, the administration of vincamine caused considerable inhibition in the gene expression and protein level of *NF-kB* and mRNA levels of *TNFα*, *IL-6*, *IL-1β*, and *PDGF* genes and up-regulation in the *PPARγ* and *klf6* genes expression as well as *PPARγ* protein level, suggesting the inhibition of the NF-kB-mediated inflammatory signaling pathway leading to the hepato-protective anti-inflammatory activity of vincamine. Furthermore, vincamine increased the activity of the PPARγ target candidates, catalase and HO-1, resulting in the activation to the hepatic anti-inflammatory and antioxidant defense mechanisms.

Furthermore, ANIT hepatotoxicity was reported to contribute to glutathione depletion; ANIT can bind to glutathione and form a reversible S-conjugate which is crucial in shuttling ANIT into bile and further initiate a group of toxic compounds (Roth and Dahm 1997). Additionally, neutrophils, which are activated during ANIT induction, release cytotoxic proteases leading to damage of target cells (Mehendale et al. 1994). Also, it was indicated that oxidative stress condition is associated with hepatic cholestasis (Copple et al. 2010).

In the present study, we evaluated the oxidative stress condition and the antioxidant status of liver tissue during the intrahepatic cholestasis induced by ANIT with and without vincamine treatment. Data in the present study revealed the hepatic suppression of SOD activity and reduction in the GSH level during the intrahepatic cholestasis, which is the antioxidant defense mechanism system of the cell, whereas an elevation of the hepatic MDA level was also observed, which is a key biomarker for assessing oxidative stress status (Singh et al. 2014). Notably, administration of vincamine, during the intrahepatic cholestasis induced by ANIT, improved the hepatic antioxidant status and modulated the oxidative stress condition; SOD activity and GSH level were elevated, while MDA level was suppressed, suggesting the hepato-protective antioxidant activity of vincamine in ANIT-induced hepatic cholestasis.

Moreover, we investigated the role of PI3K/Akt pathway. Numerous studies reported that PI3K/Akt pathway is responsible for the activation and release of nuclear factor erythroid-2 (Nrf2) dependent antioxidant system (Wang et al. 2008, 2015; Liao et al. 2019). Also, it was indicated that the inactivation of PI3K/Akt pathway is related to the toxicity of ROS; when Akt is present in the unphosphorylated condition, it could cause mitochondrial dysfunction and target bcl2 family (Zeng et al. 2011). Mitochondrial dysfunction would lead to the induction of caspases signaling cascade and the activation of apoptosis. Additionally, Akt can phosphorylate and inactivate pro-apoptotic factors, indirectly contributing to cellular protection against oxidative damage (Luo et al. 2013). Our findings revealed hepatic inhibition of PI3K/Akt pathway and reduction in bcl2 levels and the elevation in hepatic cleaved caspase 3, p53, and bax levels during the intrahepatic cholestasis induced by ANIT, which further suggest the activation of oxidative stress condition and induction of the apoptotic pathways. Interestingly, vincamine modulated these levels; it activated the PI3K/Akt antioxidant pathway and suppressed the apoptotic process through the downregulation of cleaved caspase 3, p53, bax and the elevation of bcl2 level.

Furthermore, our histopathological studies confirmed our previous analysis, whereas ANIT-induced cholestasis caused vacuolar degeneration of hepatocytes and vincamine administration improved these abnormal alterations resulting in almost normal hepatocytes and central vein with scanty inflammatory cells in the microphotographs, confirming the hepato-protective effect of vincamine in ANIT-induced hepatic cholestasis.

## Conclusion

In conclusion, vincamine alleviated hepatic dysfunction during ANIT-induced intrahepatic cholestasis. It enhanced the liver function tests and exerted hepato-protective action via its anti-inflammatory activity through the inhibition of NF-kB-mediated inflammatory signaling pathway with modulation of *PDGF, klf6,* and *PPARγ* mRNA levels. In addition, it activated the antioxidant PI3K/Akt signaling pathway with improvement in the activities of SOD, catalase, and HO-1 and the levels of GSH and MDA. Moreover, vincamine showed antiapoptotic activity through the inhibition of p53/bax/caspase 3 pathway and the elevation of bcl2 level.

## Data Availability

All data generated or analyzed during this study are included in this published article and its supplementary information files.
